# Pro- and Synbiotics to Prevent Sepsis in Major Surgery and Severe Emergencies 

**DOI:** 10.3390/nu4020091

**Published:** 2012-02-17

**Authors:** Stig Bengmark

**Affiliations:** 1 Department of Surgery, Lund University, Lund, Sweden; Email: stig@bengmark.se Tel./Fax: +44-20-7511-6841; 2 Division of Surgery and Interventional Science, University College London, 74 Huntley Street, London WC1E 6AU, UK

**Keywords:** probiotics, microbiota, nutrition, sepsis, pancreatitis, encephalopathy

## Abstract

Septic morbidity associated with advanced surgical and medical treatments is unacceptably high, and so is the incidence of complications occurring in connection with acute emergencies such as severe trauma and severe acute pancreatitis. Only considering the US, it will annually affect approximately (app) 300 million (mill) of a population of almost one million inhabitants and cause the death of more than 200,000 patients, making sepsis the tenth most common cause of death in the US. Two major factors affect this, the lifestyle-associated increased weakness of the immune defense systems, but more than this the artificial environment associated with modern treatments such as mechanical ventilation, use of tubes, drains, intravascular lines, artificial nutrition and extensive use of synthetic chemical drugs, methods all known to reduce or eliminate the human microbiota and impair immune functions and increase systemic inflammation. Attempts to recondition the gut by the supply of microorganisms have sometimes shown remarkably good results, but too often failed. Many factors contribute to the lack of success: unsuitable choice of probiotic species, too low dose, but most importantly, this bio-ecological treatment has never been given the opportunity to be tried as an alternative treatment. Instead it has most often been applied as complementary to all the other treatments mentioned above, including antibiotic treatment. The supplemented lactic acid bacteria have most often been killed already before they have reached their targeted organs.

## 1. Introduction: A Pandemic of Critical Illness

Advanced surgical and medical treatments, as well as medical and surgical emergencies, are, despite some breath-taking advances in medico-pharmaceutical and surgical treatment, still accompanied by an unacceptably high morbidity and also mortality. Worse than this, morbidity and mortality in these conditions is fast increasing and has done so all over the world for several decades. Apart from the suffering associated with this, it is also extremely costly to the individual and the health care system.

Dominating among the treatment-induced morbidities is sepsis due to bacterial, fungal or viral infections. Recent observations in the US suggest that not only the incidence, but also the severity of sepsis, has significantly increased during the last decades [1,2,3]. Especially advanced surgery carries a high rate of septic morbidity and especially esophageal, pancreatic, and gastric procedures are particularly known to represent great risk for the development of sepsis, but thoracic, adrenal, and hepatic procedures are those that have the highest sepsis-induced mortality [1,2]. It is also documented in the literature that elderly patients, men, and nonwhites are the most likely to develop sepsis as a complication to surgical treatment [1,2]. Sepsis is by far the most common medical and surgical complication, and estimated only in the US to annually affect as many as 751,000 [4,5], and cause death of app 215,000 patients (29% of treated patients) [5], making sepsis the tenth most common cause of death in this country.

## 2. Sepsis: Often a Product of So Called Auxiliary/Supportive Measures

Post-surgical morbidity and due to septic manifestations is only partly attributed to the surgeon and the surgical technique. Increasing evidence suggests instead that it is the patient’s ability to resist disease/immune defense and especially supportive measures during and around the treatment, such as mechanical ventilation, use of implants, drains and intravascular lines, but also choice of content and routes to provide nutrition, blood transfusions, choice of anesthesia and prescription of drugs, also antibiotics and immune-suppressives, that are the largest contributors to the development of septic manifestations. 

Mechanical ventilation in association with management of emergencies and surgical procedures has in recent years received increasing attention as a major contributor to chest-infections, but also to general and localized septic manifestations in the body; according to a recent study applied in the US in about 800,000 individuals every year. It is estimated to represent no less than 12% of total hospital costs, no less than $27 billion in the US alone [6]. This treatment is responsible for, not only a disproportional amount of resources used, but also for the unacceptably high morbidity and mortality associated with the treatment, especially in elderly people [6]. 

A main contributor to intensive care unit (ICU)-associated sepsis is also artificial nutrition, both enteral and parenteral; catheter-related sepsis is reported to occur in about 25% of patients fed via intravenous feeding-tubes [7]. Numerous drugs used in the ICUs including antibiotics are known to derange the immune functions, impair macrophage functions, bactericidal efficacy as well as production and secretion of cytokines. For example, supply of an antibiotic, Mezlocillin (Bayer, 150 mg/kg body weight) is demonstrated to significantly suppress essential macrophage functions and derange chemiluminescence response, chemotactic motility, bactericidal and cytostatic ability and impair lymphocyte proliferation [8]. Other common perioperative practices like use of artificial feeding regimens, preoperative antibiotics [9], and mechanical bowel preparation [10,11] will also, according to recent studies, instead of the expected prevention as expected, contribute to increased rates of treatment-associated infections. 

## 3. Gut and Chest Infections Dominate

Most of the surgical infections originate in areas with the most exposure to the exterior world, *i.e.*, the gut (63%) and chest (20%) and are pure gram-negative (22%), pure gram-positive (15%), mixed (23) or fungal (8%) [12]. It is also here that the dominating parts of the immune functions are localized: between 70–80% of the Ig-producing immunocytes of the body are found in the gut [13]. It was Marshall and his group who, in 1993, directed attention to the gut as a special source of surgical sepsis and especially to its most severe form—multiple organ failure (MOF) [14]. They observed that the most common organisms to cause ICU-acquired infections—*Candida*, *Streptococcus faecalis*, *Pseudomonas*, and coagulase-negative *Staphylococci*—were also the most common species that seemed to colonize especially the proximal GI tract. They reported that this colonization correlated well with development of invasive infection within one week: *Pseudomonas* (90% *vs.* 13% in non-colonized patients, *p* < 0.0001) and *Staphylococcus epidermidis* (80% *vs.* 6%, *p* < 0.0001). As a consequence of these observations, they coined the phrase: “the gut—the undrained abscess”. Studies during subsequent years focused mainly on microbial translocation as the major course of sepsis. 

Important observations had, however, been made by Baue, Faist and their groups already ten years earlier, and their finding that a significant portion of patients, who develop multiple organ dysfunction syndrome (MODS), and do not have an identifiable infection [15], should have a great impact on future understanding of treatment and emergency-associated sepsis. It was Goris and his collaborators, who, in 1986, based on observations like those of Baue and Faist, but also after extensive studies in animals, suggested inflammation precedes septic manifestations and an “auto-destructive inflammatory response” with or without bacterial infection, is a major cause of this severe condition [16]. 

## 4. Preceding Uncontrolled Exuberant Systemic Inflammation

Patients who develop severe septic complications are known to respond to physical and mental stress with an early exuberant acute, or chronic, super-inflammation, with signs of exaggerated and prolonged release of pro-inflammatory cytokines such as interleukin-6 (IL-6), acute phase proteins such as C-reactive protein, and plasminogen activator inhibitor 1 (PAI-1)—see [17]—a reaction strongly associated with subsequent severe exacerbation of disease, including acute respiratory distress syndrome (ARDS), and MOF. Among the observed changes associated with an exuberant inflammation in the early nervous phase are: augmented endothelial adhesion of polymorphonuclear (PMN) cells, increased production of intracellular adhesion molecule-1 (ICAM-1), priming of the PMNs for an oxidative burst, release of pro-inflammatory platelet activating factor (PAF), and a delay in PMN apoptosis [18]. Visceral adipocytes are, compared to subcutaneous fat cells, known to secrete per gram tissue much more of free fatty acids but also about three times as much IL-6, and PAI-1; observations that well explain the high risk of both chronic and acute diseases in individuals with visceral obesity [19]. The stress-induced load of these and other proinflammatory and procoagulant molecules on organs, such as the lung and the liver, can vary a thousand times or more, as the amount of fat in the abdomen can vary from a few milliliters in a lean subject to about six liters in gross obesity [20]. 

## 5. Mental and Physical Stress Potentiates the Response

An increase in growth of Gram-negative bacteria of up to 100,000 times (5 logs of order) has been demonstrated in animals exposed to noradrenaline; see Lyte’s review [21]. Old observations suggest a strong and significant association between higher blood levels of noradrenaline and adrenaline and development of severe septic conditions [22]. Luminal release of noradrenaline is a documented strong inducer of virulence of luminal bacteria [23], and much suggests that potentially pathogenic microorganisms (PPMs), normally indolent colonizers, under stress change their phenotype and become life-threatening pathogens [24]. These observations are particularly interesting as classical studies published already in the 1940s demonstrated that a 10,000 times lower dose of *Clostridium welchii* is needed to induce death in gas-gangrene in animals, when also adrenaline is administered to the animals [25]. Most likely similar potentiating effects exist when stress and stress-hormones are applied also to other pathogenic bacteria. 

## 6. Deranged and Dysfunctioning Microbiota

Microbiota is significantly reduced in Westerners. A study published in the US in 1983 reported that *Lactobacillus plantarum*, a dominating lactic acid bacterium (LAB) among plant eaters, is found in only about 25% of omnivorous Americans and in about two thirds of vegetarian Americans [26]. A more recent Scandinavian study suggest that also in healthy individuals, the most common colonic LAB are present in only half or less of the individuals: *Lactobacillus plantarum* in 52%, *Lactobacillus rhamnosus* in 26% and *Lactobacillus paracasei* ssp. *paracasei* 17% [27]. Western lifestyle with frequent mental and physical stress, and eating of processed foods with high content of saturated fats and trans-fatty acids, lack of dietary fibers, containing chemicals and pharmaceuticals, but also lack of microorganisms content in food, will significantly reduce the microbiota both with regard to diversity and extent of existing flora. Recent studies report that in Westerners, a significant shift in balance from gram-positives to gram-negatives, and subsequent increase in production of endotoxins/lipopolysaccharide (LPS) [28]. Magnesium (Mg) requirements are known to be much higher for Gram+ than for Gram− bacteria and Mg deficiency, common in Westerners, was recently shown to induce decreased content in the gut especially of *Bifidobacteria*, to increase colonic and systemic inflammation, and increase intestinal permeability. These observations are of particular interest as Mg deficiency is associated with systemic inflammation and common metabolic disorders such as type 2 diabetes, metabolic syndrome, dyslipidemia, and hypertension [29], but also observed in ICU patients. Mg-deficient patients, in comparison to patients with normal magnesium levels, are reported to exhibit a higher prevalence of severe sepsis and septic shock (57 *vs.* 11%, *p* < 0.01), a longer ICU stay (15.4 ± 15.5 *vs.* 2.8 ± 4.7 days, *p* < 0.01), and a higher mortality rate [30].

A several log-fold decrease in the commensal bacteria like *Bifidobacteria* and *Lactobacilli* are reported in trauma patients in parallel to increased populations of pathogenic bacteria such as *Pseudomonas aeruginosa* and *Staphylococcus aureus* [31]. The Western lifestyle-induced derangement of the microbiota is associated with increased permeability of intestinal mucosa, increased LPS absorption, increased endotoxemia, exaggerated inflammation, and strongly associated with metabolic disorders such as obesity and diabetes [32]—conditions, which also are commonly associated with increased surgical morbidity, surgical infections and MOF.

## 7. Overreacting Neutrophils

Severe trauma, major surgery and severe sepsis will, parallel to a significant decrease in lymphocytes, induce a significant, sometimes disproportionate, increase in circulating and tissue neutrophils, and be accompanied by persistent decline in T-4 helper lymphocytes and elevation of T-8 suppressor lymphocytes [33]. It is suggested that a T-4/T-8 lymphocyte cell ratio of <1 is a sign of severe immunosuppression and predictor of complication, such as multiple organ dysfunction syndrome, myocardial infarction, acute pancreatitis, multiple severe trauma and chemotherapeutic treatment, especially in oncology patients [34]. An early and large increase in circulating neutrophils is accompanied by tissue infiltration of neutrophils, and responsible for common posttrauma/postoperative dysfunctions such as paralytic ileus [35,36], bone marrow suppression, endothelial cell dysfunction, and to lead to tissue destruction and organ failure, particularly in lungs [37,38,39], intestines [40], liver [41] and kidney [42]. Neutrophil infiltration of distant organs [16], especially the lungs [37], is a characteristic finding in patients dying of sepsis. The extent of neutrophil infiltration is significantly aggravated by mechanical therapeutic efforts such as handling of the bowels during operation [35], and ventilation of the lungs [43]. Poor nutritional status, preexisting immune deficiency, obesity, diabetes and high levels of blood sugar [44] contribute to immune deterioration and to increased expressions in the body of molecules such as NF-κB, COX-2, LOX and iNOS [45,46]. The disproportionate increase in circulating neutrophils is to a great extent inhibited by supplementation of antioxidants [47,48] and specific probiotics [49]. 

**Figure 1:**
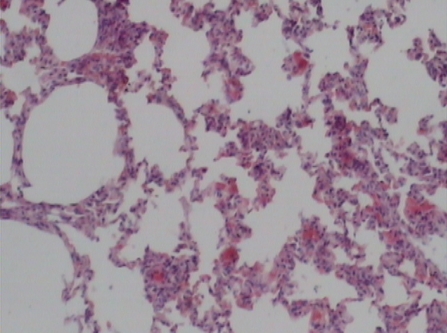
Control group.

**Figure 2:**
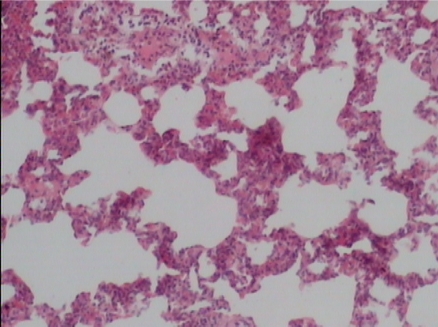
Synbiotic treated group.

**Figure 3:**
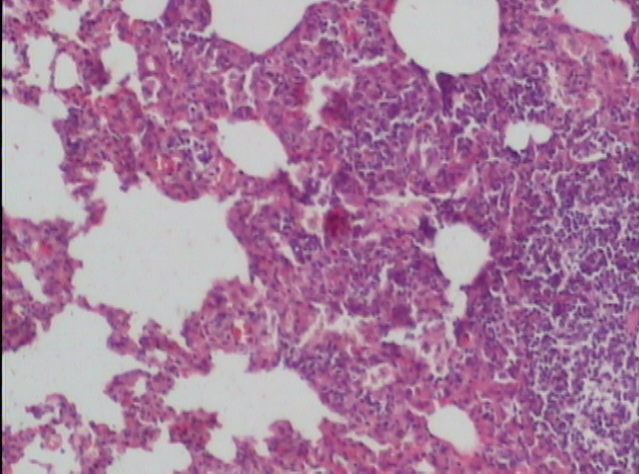
Prebiotic group.

Supplementation of probiotics will effectively prevent neutrophil infiltration of the lung and also reduce the subsequent tissue destruction, as demonstrated in studies with inflammation induced by cecal ligation and puncture (CLP). A synbiotic formulation, Synbiotic 2000 Forte (see further below), was administered orally before the induced trauma and effectively prevented both neutrophil accumulation and tissue destruction in the lungs [50]. Most interestingly, these effects were obtained also when the LAB of the composition were injected subcutaneously (Figure 1,Figure 2,Figure 3) [51]. 

The average neutrophil count in the lungs (average of five fields) was: mixture of LAB and bioactive fibers 9.00 ± 0.44 (1), only LAB 8.40 ± 0.42 (2), only bioactive fibers 31.20 ± 0.98 (3), placebo (non-fermentable fiber) 51.10 ± 0.70 (4). The reduction of inflammation by the treatment was also demonstrated by significant reductions in myeloperoxidase (MPO), malondialdehyde (MDA), and nitric oxide (NO): MPO being 25.62 ± 2.19 (1), 26.75 ± 2.61 (2), 56.59 ± 1.73 (3), and 145.53 ± 7.53 (4) respectively (resp.), MDA 0.22 ± 1.31 (1), 0.28 ± 3.55 (2), 0.48 ± 5.32 (3) and 0.67 ± 2.94 (4) resp. and NO 17.16 ± 2.03 (1), 18.91 ± 2.24 (2), 47.71 ± 3.20 (3) and 66.22 ± 5.92 (4) resp.—all differences being statistically significant (>0.05).

## 8. Personal Experience with Pro- and Synbiotics

My interest in microbiota and probiotics stems back to 1986. Since 1963, I was a surgeon focusing my interest on liver surgery, and was actively searching for new tools to combat the unacceptably high rate of perioperative infections associated with major surgery and particularly with extensive liver resections. A review of our last 81 liver resections yielded some unexpected information, which directed my interest to human microbiota and probiotics. It was standard practice at that time, in connection with surgery, to provide the patients with an antibiotic umbrella for at least five days with the hope that this treatment might somewhat reduce the rate of infections. The shocking information from this study was that only 57/81 patients had in fact received this treatment, while the treatment had been neglected in as many as 24/81 patients [52,53]. However, the unexpected information from this review was that there were no cases of sepsis reported in the patients who had not received prophylactic antibiotics. As a matter of fact, all manifestations of sepsis had occurred in the antibiotic-treated patients. A growing awareness of the importance of human microbiota [54] and the eventual possibility of reconditioning of the gut through probiotic treatment [55] was at that time visible. It was also increasingly understood that lifestyle, chemicals and pharmaceuticals, in addition to the disease *per se*, could impair the microbiota and immune defense. 

It seemed likely that powerful probiotic treatment could constitute an effective prevention of unwanted infections in disease and also in surgical and emergency medical cases. That was the reason why I established collaboration with experts in microbiology, chemistry, nutrition, experimental and clinical science to seek opportunities find, develop and test, experimentally and clinically, probiotics, which could be expected to be powerful tools to prevent sepsis. Our first efforts resulted in a one LAB/one fiber composition, produced by fermentation of oat meal with *Lactobacillus plantarum* strain 299 [56,57,58]. I worked with this synbiotic composition for more than ten years. This formula is produced and marketed by Probi AB, Lund, Sweden.

After 1999 and until today, I have continued my research with a four LAB/four fiber composition, which consists of a mixture of 10^10^ (standard version—Synbiotic2000™) and a mixture of 10^11^ (forte version—Synbiotic 2000 forte™) of four different LAB: *Pediococcus pentosaceus* 5-33:3, *Leuconostoc mesenteroides* 32-77:1, *Lactobacillus paracasei* subsp paracasei 19, and *Lactobacillus plantarum* 2362 combined with 10 g of fibers; 2.5 g of each of four fermentable fibers: betaglucan, inulin, pectin and resistant starch [59,60]. The standard Synbiotic 2000 contains thus 4 × 10 billion LAB = 40 billion and the stronger version, Synbiotic 2000 forte, 4 × 100 billion LAB = 400 billion resp. of lactic acid bacteria. The formula is marketed by Ionian Pharma, Glyka Nera, Greece and Synbiotic AB, Höganäs, Sweden. 

### 8.1. Early Treatment in Major Trauma

Two prospective randomized trials with Synbiotic 2000 and Synbiotic 2000 Forte respectively have been concluded. The first study compared in-patients with acute extensive trauma receiving four types of treatment: (1) Synbiotic 2000 (40 billion LAB/day); (2) A soluble fiber; (3) A peptide diet (Nutricomp, Braun Inc. Germany); and (4) Glutamine supplementation. Treatment with Synbiotic 2000™ led to a highly significant reduction in number of chest infections (4/26 patients—15%) compared to peptide diet (11/26 patients—42%, *p* < 0.04), glutamine treatment (11/32 patients—34%, *p* < 0.03) and only fiber treatment (12/29 patients—41%, *p* < 0.002) [61]. Also the total number of infections was significantly decreased: Synbiotic 2000™ 5/26 patients (19%), peptide 13/26 patients (50%) glutamine16/32 patients (50%) and only fibers 17/29 patients (59%). 

In another study, 65 polytrauma patients were randomized to receive either Synbiotic 2000 Forte (400 billion LAB + 10 gram of fibers, see above) or maltodextrine as placebo, once daily, for 15 days. Significant reductions were observed in number of deaths (5/35 *vs.* 9/30, *p* < 0.02), severe sepsis (6/35 *vs.* 13/30, *p* < 0.02), chest infections (19/35 *vs.* 24/30, *p* < 0.03), central line infections (13/32 *vs.* 20/30, *p* < 0.02), and ventilation days (average 15 *vs.* 26 days [62]). A total of pathogenic microorganisms of 54 were cultivated in the Synbiotic treated group compared to 103 in the fiber-only group [63,64]. Repeat analyses also revealed that serum levels of endotoxin (lipopolysaccharide (LPS)) were decreased and time to bloodstream infection significantly prolonged in patients treated with Synbiotic 2000 forte.

### 8.2. Early Treatment in Severe Acute Pancreatitis

Patients with severe acute pancreatitis were randomized to receive, during the first 7 days daily administered through a nasojejunal tube, either a freeze-dried preparation containing live *Lb plantarum* 299 in a dose of 10^9^ together with a substrate of oat fiber or a similar preparation, but heat-inactivated [65]. The study was concluded when, on repeat statistical analysis, significant differences in favor of one of the two groups were obtained. This occurred when a total of 45 patients had entered the study. 22 patients had at that time received treatment with live and 23 with the heat-killed *Lb plantarum* 299. Infected pancreatic necrosis and abscesses were seen in 1/22 (4.5%) in the live LAB group *vs.* 7/23 (30%) in the heat-inactivated group (*p* = 0.023). The only patient in the lactobacillus group, who developed infection, a urinary infection, did that on the 15th day, e.g., at a time when he had not received treatment for eight days. The length of stay was also considerably shorter in the live LAB group (13.7 days *vs.* 21.4 days), but the limited size of the material did not allow statistical significance to be reached.

Sixty-two patients with severe acute pancreatitis (SAP) (Apache II scores: Synbiotic 2000-treated 11.7 ± 1.9, controls 10.4 ± 1.5) were given either two sachets/day of Synbiotic 2000™ (2 × 40 billion LAB/day and totally 20 g fibers) or the same amounts of fibers (20 g) as in Synbiotic 2000™ during the first 14 days after arrival to the hospital [66]. 9/33 patients (27%) in the Synbiotic 2000-treated group and 15/29 patients (52%) in the only fiber-treated group developed subsequent infections. Eight out of 33 (24%) Synbiotic 2000-treated patients, and 14/29 (48%) of the only fiber-treated patients, developed systemic inflammatory response syndrome (SIRS), MOF or both (*p* < 0.005). A total of pathogenic microorganisms of seven were cultivated in the Synbiotic-treated group compared to 17 in the only-fiber group.

### 8.3. Effects on “Mind Clarity”: Encephalopathy

Patients with critical illness, as well as patients with chronic disorders such as liver cirrhosis and diabetes, often suffer a mild, but sometimes, severe confusion, which often has its origin in the gut [67]. Increasing evidence suggest that probiotics, alone and/or in combination with plant antioxidants and fibers, possess strong neuro-endocrine modulatory effects and alleviates effects of physical and mental stressors both early [68] as well as later in life [69]. We undertook some studies to explore the effects of synbiotic treatment in patients with liver cirrhosis and minimal hepatic encephalopathy (MHE). 

Fifty-five patients with MHE were randomized to receive, for 30 days: (1) Synbiotic 2000 (*n* = 20), (2) The fibers in the composition alone (*n* = 20), or (3) Placebo (*n* = 15). All cirrhotic patients with MHE were found to have severe derangements of the gut micro-ecology, and significant overgrowth of potentially pathogenic *Escherichia coli* and *Staphylococcal* species. Synbiotic treatment increased the fecal content of non-urease-producing *Lactobacillus* species significantly and reduced the numbers of potentially pathogenic micro-organisms. The treatment was also associated with a significant reduction in endotoxemia and in blood ammonia levels. A documented reversal of MHE was obtained in half of the treated patients; the Child-Turcotte-Pugh functional class improved in about 50% of cases [70]. Treatment with fermentable fibers alone also demonstrated substantial benefit in a proportion of patients.

Thirty cirrhotic patients were randomized in a second study to receive Synbiotic 2000 or placebo for only 7 days. Viable fecal counts of Lactobacillus species, Child-Pugh class, plasma retention rate of indocyanine green (ICGR15), whole blood tumor necrosis factor alpha (TNF-a) mRNA and IL-6 mRNA, serum TNF-a, soluble TNF receptor (sTNFR)I, sTNFRII, IL-6 and plasma endotoxin levels were measured pre- and post-treatment. The treatment with Synbiotic 2000 was associated with significantly increased fecal lactobacilli counts and significant improvements in ICGR15 and Child-Pugh class [71]. Significant increases in whole blood TNF-a mRNA and IL-6 mRNA, along with serum levels of sTNFRI and sTNFRII, were also observed and TNF-a and IL-6 levels correlated significantly, both at baseline and post-synbiotic treatment [71]. Synbiotic-related improvement in ICGR15 was significantly accompanied by changes in IL-6, both at mRNA and protein levels, but was unrelated to levels of plasma endotoxin. No significant changes in any parameter were observed following placebo treatment. Even this study concluded that short-term synbiotic treatment modulates gut flora significantly and improves liver function in patients with cirrhosis. 

## 9. Studies by Others

In a recent study twenty-nine SIRS patients with a serum C-reactive protein (CRP) level above 10 mg/dL, received a synbiotic composition consisting of *Bifidobacterium breve* and *Lactobacillus casei*, in combination with galactooligosaccharides. The incidence of infectious complications such as enteritis, pneumonia, and bacteremia were (compared to historical controls) significantly lower in the treated group [72]. Analysis of fecal flora demonstrated significantly higher levels of *Bifidobacteria* and *Lactobacillus*, and also of total organic acids, particularly short-chain fatty acids. 

## 10. Studies with No, or Adverse, Effects

### 10.1. Ecologic 641™

Two hundred and ninety eight patients with predicted severe acute pancreatitis and with APACHE II score ≥8, Imrie score > 3, or C-reactive protein >150 mg/L) within 72 h of onset of symptoms were included in a multicenter, double-blind, placebo-controlled trial, randomly assigned to receive either a multispecies probiotic preparation (*n* = 153) or placebo (*n* = 145), administered enterally twice daily for 28 days. The probiotic composition supplemented, Ecologic 641 (Winclove Bio Industries, Amsterdam, The Netherlands), consisted of 10^10^ of each of six different strains of freeze-dried, viable bacteria: *Lactobacillus acidophilus*, *Lactobacillus casei*, *Lactobacillus salivarius*, *Lactococcus lactis*, *Bifi dobacterium bifidum*, and *Bifidobacterium lactis*. It contained also cornstarch and maltodextrins. The subsequent analyses were based on 152 individuals in the treated group and 144 in the placebo groups. Infectious complications occurred in 46 (30%) of patients in the probiotics group and in 41 (28%) of those in the placebo group (relative risk 1.06, 95% CI 0.75-1.51). Twenty-five (16%) patients in the probiotics group died, compared to nine (6%) in the placebo group (relative risk 2.53, 95% CI 1.22-5.25) [73]. Furthermore, nine patients in the probiotics group developed bowel ischemia, eight of which with fatal outcome, compared to none in the placebo group (*p* = 0.004).

### 10.2. *Lactobacillus plantarum* 299™: ProViva™

One hundred and three critically ill patients were randomized to receive 1 ProViva, a fruit drink containing 5% of LAB-fermented oat and live *Lactobacillusb plantarum* 299v with a density of 5 × 10^7^ (*n* = 52) or conventional nutrition therapy alone (*n* = 51). The treatment demonstrated no identifiable effect in terms of bacterial translocation (12% *vs.* 12%; *p* = 0.82), gastric colonization with enteric organisms (11% *vs.* 17%, *p* = 0.42), or septic morbidity (13% *vs.* 15%; *p* = 074), serum CRP levels or mortality [74]. In another study, 11 patients undergoing elective abdominal surgery during a median time of 9 days (range 5-18 days) received lactobacillus 299v (ProViva) to total average amounts of 3250 mL (range 2100-9000 mL), and were compared to 11 control patients. The authors found no significant differences between the ProViva group and the control group in terms of concentrations of plasma cells, IgA positive cells or IgM positive cells in the lamina propria [75]. There was a significantly higher concentration of IgM at the mucosal surface in the control group (*P* = 0.02, Fishers Exact test mid P), but no difference in terms of IgA. 

### 10.3. *Lactobacillus rhamnosus* GG™

Sixty-one patients were in a study in pediatric ICU, randomized: 31 patients to receive treatment with one capsule of *Lactobacillus rhamnosus* strain GG in a dose of 10^10^, and 30 patients to receive one capsule with insulin daily (control group). No difference in rate of infections was observed between the groups; the mean number of infections in the treatment and control groups was 1.83 and 1.33, respectively. 9/31 patients in the probiotic-treated group developed in total 15 nosocomial infections: six bloodstream infections (40%), five tracheo-bronchitis (33%), two pneumonia (13%), and two UTI (13%) [76]. There were totally six deaths during the study period; four in the placebo group and two in the treatment group. No cases of *Lactobacillus* bacteremia or other serious adverse effects were observed. 

### 10.4. Synbiotic 2000™/Synbiotic 2000 Forte™

Synbiotic 2000 (40 billion LAB) was given daily to 162 patients and 168 control patients received similarly only the fibers of the synbiotic composition. No significant differences in demographics, mortality (ICU or hospital), morbidity, maximal multiple organ dysfunction (MOD) score, ICU length of stay, diarrhea days or septic complications were observed between intervention and control groups [77]. Similarly *post hoc* analysis of those patients who received at least 75% of prescribed doses revealed no difference in ICU mortality (21% *vs.* 20%) or hospital mortality (32% *vs.* 26%) between the intervention and control groups. It was especially noted that no episodes of intestinal ischemia were observed in either group.

Two hundred and fifty nine enterally fed critically ill patients, expected to require mechanical ventilation for 48 h or more were enrolled in a study; 130 patients received Synbiotic 2000 FORTE^®^ (twice a day) and 129 patients a cellulose-based placebo for a maximum of 28 days. The oropharyngeal microbial flora and colonization rates were unaffected by the synbiotic treatment. The overall incidence of ventilator associated pneumonia (VAP) was lower than anticipated (11.2%) and no statistical difference was demonstrated between the groups receiving synbiotic or placebo; incidence of VAP (9 and 13%, *P* = 0.42), VAP rate per 1000 ventilator days (13 and 14.6, *P* = 0.91) or hospital mortality (27 and 33%, *P* = 0.39), respectively [78]. No negative effects of the treatment were observed. 

### 10.5. Trevis™

A total of 90 patients admitted to an ICU were randomized to receive either synbiotic or placebo (45 into each group). The synbiotic treatment consisted of supplying, three times a day, a capsule of Trevis^TM^ (Chr Hansen Biosystem, Denmark), containing 4 × 10^9^ colony forming units of each of *L. acidophilus* La5 (La5), *B. lactis* Bb-12 (Bb-12), *S. thermophilus* and *L. bulgaricus*. In addition the prebiotic oligofructose (7.5 g of RaftiloseTM powder, Orafti Active Food Ingredients, Belgium) was administered twice a day. The patients in the synbiotic group demonstrated after 1 week of therapy significantly lower incidence of potentially pathogenic bacteria ( *vs.* 75%, *P* = 0.05) and multiple organisms (39% *vs.* 75%, *P* = 0.01) in their nasogastric aspirates than the controls [79]. However, there were no significant differences between the groups in terms of intestinal permeability, septic complications or mortality. 

### 10.6. VSL#3™

Twenty-eight patients were enrolled and randomly assigned to one of three treatment groups: (1) placebo (*n* = 9); (2) viable probiotics—2 sachets daily of VSL#3™ (*n* = 10); or (3) bacterial sonicates—not viable VSL#3 bacteria (*n* = 9). Each sachet of the supplemented probiotic, VSL#3 (VSL Pharmaceuticals, Ft Lauderdale, FL) contains 900 billion viable lyophilized bacteria of 4 strains of *Lactobacillus* (*L. casei*, *L. plantarum*, *L. acidophilus*, and *L. delbrueckii* subsp. *bulgaricus*) plus 3 strains of *Bifidobacterium* (*B. longum*, *B. breve*, and *B. infantis*), plus *Streptococcus salivarius* subsp. *Thermophiles*, totally eight strains. Intestinal permeability decreased in all treatment groups. The rate of severe sepsis and MODS were not significantly affected by the treatment, although a significantly larger increase in systemic IgA and IgG concentrations were observed in the group supplied with live bacteria than in the patients who received placebo or sonicated bacteria (*P* < 0.05) [80]. 

## 11. Why do Studies Fail?

Critical care units are in general a highly artificial environment and the burden of environment-induced physical and mental stress and subsequent status of systemic hyper-inflammation on the patient is unbearable. Patients treated under these conditions are in many aspects dysfunctional, the whole microbiota is gone and probiotic bacteria supplied will most often be killed already before they have reached its target organ. This artificiality seems to vary from country to country, and sometimes also from hospital to hospital, observations that might explain the great variation in outcome from studies undertaken in different regions and countries. 

Probiotic treatment has never been given the chance as an alternative treatment; it has only been tried as a treatment complementary to all the other standard treatments. As discussed above, a series of auxiliary measures and particularly mechanical ventilation [7] and treatment with various drugs, including antibiotics [8,9], but also clinical nutrition solutions belong to those factors which promote super-inflammation and, indirectly, infection. Enteral nutrition formulas are also known to induce loss of intestinal barrier function, promotes bacterial translocation, and impairs host immune defense [81], a phenomenon, observed in humans and further elucidated in animal studies. The incidence of bacterial translocation to the mesenteric lymph node was in such studies significantly increased when the animals were fed nutrition formulas such as Vivonex (53%), Criticare (67%), or Ensure (60%) (*p* < 0.05) [82,83,84]. Similar observations have also been made in patients. Significant elevations in pro-inflammatory cytokines were observed in patients, who after pancreatoduodenectomy are fed a standard enteral nutrition solution (Nutrison): IL-1beta—day 7 (*P* < 0.001), day 14 (*P* = 0.022); TNF-alpha—day 3 (*P* = 0.006), day 7 (*P* < 0.001) [85]. Of special interest are the observations that such changes were not observed when the standard nutrition was replaced with a formula that claimed to have immune-modulatory effects (Stresson). Instead anti-inflammatory cytokines were seen significantly elevated: IL-1ra/s: day 7 (*P* < 0.001), IL-6: day 10 (*P* = 0.017), IL-8: day 1 (*P* = 0.011) days 3, 7, 10, and 14 (*P* < 0.001) and IL-10: days 3 and 10 (*P* < 0.001).

The type of bacteria to be chosen for probiotic purpose is also critical. Only a few LAB strains have demonstrated ability to influence the immune system, reduce inflammation and/or eliminate or reduce unwanted pro-inflammatory molecules from foods. Even strains, which carry sometimes the same name have often different and sometimes opposite effects. A recent study selected 46 strains of *Lactococcus lactis* from about 2600 LAB and compared their ability to induce cytokines. It was demonstrated that the inter-strain differences in ability to produce pro- and anti-inflammatory cytokines were great [86], an observation which underlines the importance of extensive animal and preclinical studies before a LAB or combination of LAB be chosen as probiotic.

## 12. Choice of Lactic Acid Bacteria as Probiotics

Especially desirable as probiotics are strains that improve immune function by increasing the number of IgA-producing plasma cells, improve phagocytosis, and influence the proportion of Th1 cells and NK cells [87]. Among the strains with a strong anti-inflammatory record are *Lactobacillus paracasei* subsp. *paracasei*, *Lactobacillus plantarum*, and *Pediococcus pentosaceus*. Especially *Lactobacillus paracasei* seems to have a solid record. It has been shown to induce cellular immunity, stimulating the production of suppressive cytokines such as TGFβ and IL-10; to suppress Th2 activity, CD4 T-cells [88,89], and splenocyte proliferation [90]; and to decrease antigen-specific IgE and IgG1 [91]. *Lactobacillus paracasei* was shown to be the strongest inducer of Th1 and repressor of Th2 cytokines when more than a hundred LAB strains were compared [92]. A recent study in rats compared the ability of four different strains: *Lactobacillus paracasei*, *Lactobacillus johnsonii*, *Bifidobacterium longum*, or *Bifidobacterium lactis* to control *Trichinella spiralis*-induced infection. *Lactobacillus paracasei*, but none of the others, were able to reduce infection-associated Th2 response, muscle levels of TGF-β, COX-2 and PGE2 and to attenuate infection-induced muscle hyper-contractility [93]. Another recent study compared the ability to reduce stress-induced changes in gut permeability and sensitivity to colorectal distension of three probiotic strains: *Bifidobacterium lactis* NCC362, *Lactobacillus johnsonii* NCC533, and *Lactobacillus paracasei* NCC2461. *Lactobacillus paracasei* but none of the other LAB, significantly restored normal gut permeability, and reduce visceral hyperalgesia and visceral pain [94]. 

*Lactobacillus plantarum* has also an excellent record. When the ability of fifty different LAB to control twenty-three different *Clostridium difficile* (C diff) strainswere studied, *Lactobacillus paracasei* and *Lactobacillus plantarum* were the only strains with ability to effectively eliminate all *C. diff* strains—more than half of the tried LAB strains were totally ineffective, and some only against a few [95]. Some LAB can be potentiated in their efficacy by simultaneous supply of prebiotic fibers (probiotics + prebiotics ≥ synbiotics). However, there are great differences in the ability of different strains to ferment and utilize plant fibers, especially when it comes to semi-fermentable fibers such as oligofructans. Only a handful of LAB from the 712 tested strains demonstrated in a study ability to ferment inulin and phlein, namely: *Lactobacillus plantarum* (several strains), *Lactobacillus paracasei* subsp. *paracasei*, *Lactobacillus brevis* and *Pediococcus pentosaceus* [96].

## 13. Discussion: The Future “Designer Probiotics”?

Two recent studies provide a fascinating insight into the future. Gene expression of human duodenal mucosa cells were studied after exposure to one of the following four lactic acid bacteria: *Lactobacillus plantarum* WCFS1 [97], *Lactobacillus acidophilus* L10, Lactobacillus *casei* CRL-431 and *L. rhamnosus* GG [98], administered in a cross-over study to healthy volunteers in a dose of 10^10^. Mucosal biopsies were taken from duodenum after 6 h and compared to control biopsies. The interventions did not impair immune and metabolic homeostasis. Nevertheless, a fascinating and most distinct influence on expression of several hundred genes (transcriptome) was reported after administration of each of the LAB. For seemingly the first time, different probiotic lactobacilli are reported to induce more or less strain-specific and markedly different expression profiles, much similar to what is known to occur with ingestion of various foods, especially plant ingredients [99,100], but also similar to the effects observed after supplying certain pharmaceuticals. Thus *L. plantarum* was reported to modulate overt adaptive immune responses [97], *L. acidophilus* to suppress inflammation *, L. casei* to stimulate Th1 response and improve the Th1-Th2 balance, *L. rhamnosis* to influence cellular growth and proliferation [98]. The effects were suggested to resemble, although distinctly milder, those obtained by specific pharmaceuticals: *L. acidophilus*—antagonists of α-receptor activity, guanine antagonists, synthetic corticosteroids and flavonoids, *L. casei*—modulators of GABA receptors, cholinergic blocking agents, antagonists of β-adrenergic receptors, *L. rhamnosus*—glycoside steroids, alkaloids, protein synthesis inhibitors and protein kinase C inhibitors. A large person-to-person variation in response was also reported.

The responsiveness to ingestion of various LAB seems to be strongly influenced, not only by eventual genetic background and existing resident microbiota, but also by lifestyle, and particularly by diet, which might explain the person-to-person differences in response observed in the above studies [97,98], but also differences in outcome, when applied in critically ill patients, as reported in this review.

In the immediate future, probiotics will most likely continue to find its dominating niche as treatment given to rather healthy and health-concerned individuals with the main aim to promote health and prevent disease. However, it is likely that probiotics, as our knowledge in nutragenomics and therapeutic microbiology increases, will be generally regarded also as valuable tools for treatment of various acute and chronic diseases, also in critically ill patients. The review of studies reported to date and presented in this review supports such an assumption. Probiotics will, however, not be generally accepted and utilized as clinical tools until radical and profound changes in treatment practices in care of the critically ill and other patient groups are implemented. 
